# Growth in infants, children and adolescents with unilateral and bilateral cerebral palsy

**DOI:** 10.1038/s41598-022-05267-y

**Published:** 2022-02-03

**Authors:** Maria de las Mercedes Ruiz Brunner, Eduardo Cuestas, Florian Heinen, Andreas Sebastian Schroeder

**Affiliations:** 1 Instituto de Investigación en Ciencias de la Salud, Universidad Nacional de Córdoba, Consejo Nacional de Investigaciones Científicas y Técnicas, Bv de la Reforma S/N, Pabellón de Biología Celular, Ciudad Universitaria, CP: 5016 Córdoba, Argentina; 2 Department of Pediatric Neurology, Developmental Medicine and Social Pediatrics, University Hospital of Munich (LMU), Hauner Children’s Hospital, Munich, Germany; 3 Cátedra de Clínica Pediátrica, Hospital Nuestra Señora de la Misericordia, Facultad de Ciencias Médicas, Universidad Nacional de Córdoba, Córdoba, Argentina; 4 Ludwig Maximilian University of Munich (LMU), Hauner Children’s Hospital, Paediatric Neurology, Developmental Medicine, Lindwurmstr. 4, 80337 Munich, Germany

**Keywords:** Nutrition, Paediatrics, Public health

## Abstract

To compare growth patterns during infancy, childhood and adolescence in children with unilateral and bilateral cerebral palsy (CP) phenotype and to assess the association with gross motor impairment, dysphagia and gestational age. We retrospectively studied 389 children with CP from a single center population in Munich, Germany. 1536 measurements of height and weight were tabulated and z-scored from 6 to 180 months of age. Generalized linear mixed model were used to examine the association between growth, GMFCS, dysphagia and gestational age by CP phenotype. Children with unilateral CP tend to grow similarly to their typically developed peers. In the main effect model, bilateral CP phenotype was significantly associated with decreased mean z-scores for height (β [95% CI] − 0.953 [− 1.145, − 0.761], p < 0.001), weight (− 0.999 [− 1.176, − 0.807], p < 0.001) and BMI (β [95% CI] − 0.437 [− 0.799, − 0.075]), compared with unilateral CP phenotype. This association remained significant in the interaction models. The height-for-age z-scores, weight-for-age decreased z-scores and BMI-for-age z-scores of children with bilateral CP and GMFCS III–V or dysphagia decreased more significantly than those of children with unilateral CP. Preterm birth was not significantly associated with decreased growth in height, weight and BMI. Reduced growth in children with bilateral CP was strongly associated with moderate to severe impairment in gross motor function (GMFCS III–V) and dysphagia.

## Introduction

Cerebral palsy (CP) is a motor disability with a prevalence of 2–3‰ in the general population^1^. CP is an umbrella term, which includes a broad spectrum of different clinical phenotypes^2^. Prematurity is the highest risk factor for CP. The gestational age-specific prevalence was recently shown in a Swedish population-based study^3^. For < 28 weeks of gestational age (GA) it was 59.0 per 1000 live births, 45.7 for 28–31 GA, 6.0 for 32–36 and 1.2 for > 36 GA. Hemiplegia (unilateral CP) accounted for 44%, diplegia (bilateral spastic CP) for 34%, tetraplegia for 5% (bilateral spastic CP), dyskinetic CP for 12% and ataxia for 3%. The etiology was considered prenatal in 38%, perinatal and neonatal in 38% and remained unclassified in 24%^3^.

Children with CP are generally shorter and lighter than children with typical development (TD)^4,5^. The disparity in physical size has already been shown to be present with diagnosis at 2 years of age^5^. Growth problems increase with motor impairment and age^5,6^. Feeding difficulties resulting in undernutrition are considered an additional key factor that influences growth^5,7–11^. Moreover, in a cohort study between ages 18 months to 5 years children with CP that were born prematurely showed restrictive growth compared to their peers with CP born at term^12^. Prematurity has been associated with shorter stature in toddlers with TD^13^, but has not been fully explored in children with CP older than 5 years until adolescence.

Optimal growth during infancy principally depends on an adequate nutritional intake. Beyond infancy, growth hormones, malnutrition, environment and health related factors become progressively important^14^. Therefore, the age at which growth impairments occurs may provide clues to better understand the different variables involved in growth restriction in different subgroups of children with CP. This may help to resolve the controversy of whether CP per se leads to reduced growth or the intrinsic growth potential of children with CP is equivalent to that of children with TD.

The aim of this study was to compare growth patterns during infancy, childhood and adolescence in children diagnosed with unilateral and bilateral CP phenotype in relation to healthy peers and how additional variables such as preterm birth, eating and drinking difficulties (dysphagia) and severity of motor impairment (GMFCS Level) may affect growth.

## METHODS

### Participants

Retrospective data were collected from electronic medical records of children with CP from 6 to 180 months (15 years) of age treated at the integrated Social Paediatric Centre (iSPZ) at the Hauner Childrens Hospital, University of Munich, Germany, between January 2010 and June 2019.

All children with confirmed diagnosis of CP due to a unilateral or bilateral perinatal brain lesion (ischemia, bleeding, hypoxia, inflammation) were included. Children with brain lesions acquired later than 24 months of age, cerebral malformations due to a genetic disorder, neuro-degenerative, progressive disorder or absence of data were excluded.

Growth was compared between children with unilateral CP and bilateral CP, considering explanatory variables (GMFCS, preterm birth and dysphagia). The reference group was children with unilateral CP. The CP phenotype was defined as unilateral or bilateral according to the Surveillance of Cerebral Palsy in Europe^15^.

### Outcome variables

Growth of German children differs substantially from children in other countries. They have shown to be significantly taller, especially in the extreme percentiles when compared to the World Health Organization reference population^16^. Therefore, growth parameters of healthy German children were used as our reference population as recently published by the KiGGS Study^11^. We calculated z-scores to compare German children with CP to their TD peers. To detect relevant confounders in a multivariate analysis, we calculated height for age z-scores (i.e. length or height), weight for age z-scores and BMI z-scores^17^. The z-scores were calculated for 17 predefined age points: 6, 12 and 18 months of age, and at yearly intervals from 24 to 180 months of age respectively. In preterm children, corrected age was calculated until 2 years of age^18^.

Anthropometric measurements were carried out by qualified health professionals (pediatricians, pediatric neurologist, physiotherapist, or specialized nurses) according to standard procedures^19^. Infants were weighed undressed and children and adolescents with light clothes using an electronic scale (SECA, model 354 and 834, Hamburg, Germany). Body weight was recorded to the nearest 100 g using an electronic scale (SECA, model 799 and 877, Hamburg, Germany). Length and standing height were measure according to the child’s age and ability to stand upright, using a mechanical measuring rod (SECA, model 216, Hamburg, Germany). When the child could not stand, length was measured in the supine position. The term height is used in this study synonymously with length. When direct height could not be obtained, it was not included in the medical records. All measurements were taken twice and the average measurement was used for analysis.

### Explanatory variables

Gestational age and birth weight were collected from records during the initial visit to the iSPZ Hauner. Gestational age was calculated from the first day of the last menstrual period^17^. Prematurity is defined as babies born alive before 37 weeks of gestation. Dysphagia was retrieved from the electronic medical records. Dysphagia, a disorder characterised by difficulty in swallowing, was defined by the caring physician and neuropediatrician at the iSPZ Hauner according to ICD-10. Dysphagia was determined when the swallowing dysfunction provoked difficulty or inability to form or move the alimentary bolus safely from the mouth to the oesophagus^20^.

Gross motor function was classified according to the Gross Motor Function Classification System (GMFCS). The GMFCS, a reliable and valid method for evaluating motor function, was developed for children and adolescents with CP and establishes five levels of motor function. The GMFCS level was classified by physicians and physiotherapists according to the expanded and revised definition^21^. We dichotomized gross motor function as mild (GMFCS I–II) and moderate-severe (GMFCS III–V) motor impairments for analytical purposes.

### Statistical analysis

Data were reported in absolute (n) and relative (%) frequencies, mean ± SD, or medians with interquartile ranges (IQR) as appropriate. Differences between groups were tested using fisher’s exact test or Kruskal–Wallis test for proportions, and difference for continuous data was tested using the t-test or Mann–Whitney-*U*-test as applicable.

For the multivariate analysis, first the differences between the z-scores for anthropometric measurements and clinical covariates were examined by t-test, or Mann–Whitney-*U*-test as applicable. Variables with p < 0.05 in bivariate analysis were considered for inclusion in the linear mixed effects models.

A generalized linear mixed model (GLMM) was used to analyze longitudinal data. The analysis enables characterization and comparison of changes over time of growth expressed as mean z-score and the association with all relevant clinical covariates (GMFCS Level I&II versus III–V, dysphagia (present or absent) and prematurity (< 37 weeks of gestational age)) were performed using. GLMM allows complex models for the covariance, and can also handle unbalanced data, and accommodate continuous and categorical covariates^22^. GLMM approach accounts for the correlation induced by serial measurements on the same individual, as well as differences in the timing of the measurements and individual differences in initial status and rate of growth.

The analysis was carried out in two steps, the main effect model and the interaction model. Main effects GLMM were used to evaluate z-score changes with full adjustment (controlling for all significant covariates) including variables one by one. The reference group was children with unilateral CP. The magnitude of difference in growth gain was expressed as the β coefficient with 95% confidence intervals (CIs). The variables in the main effect model with significant difference established in p < 0.01 were included in the interaction model to evaluate the effect modification according to the potential confounders. The final models were chosen to have the best fit statistics defined by the lowest restricted maximum likelihood. In the analyses, we excluded values > 5 SD which were treated as outliers. Statistical significance in the interaction model was set at p < 0.05. All analyses were performed by using IBM SPSS statistical software, V. 25 (IBM Corp, Armonk, New York, U.S.A.).

### Ethics

The study was approved by the Institutional Review Board of the Research Ethics Commission of University of Munich (LMU) (No. 18-759), and the requirement for informed consent was waived by the ethics commission. The study was registered in the DRKS—German Clinical Trial Register (DKRS00016407). Privacy, confidentiality and security of participant’s personal data were safeguarded. All methods were performed in accordance with the relevant guidelines and regulations.

## Results

### Participants

Growth data from 459 children with CP were derived from 1628 patient visits. 21 (4.6%) children with malformations, genetic syndromes, degenerative or progressive lesions were excluded, 49 (10.7%) children were excluded due to missing data. The final sample consisted of 389 children with CP with 1536 measurements. Of these children, 226 (58.1%) were males and 163 (41.9%) were females. The number of measurements performed on each child had a median of 6 with a range from 1 to 14.

### Bivariate analysis

There were no significant differences between unilateral and bilateral CP groups in age and sex distribution. Mean height, weight and BMI at the last visit were significantly greater in children with unilateral CP than in those with bilateral CP (p < 0.001). Motor impairment, dysphagia, and prematurity were significantly more prevalent in children with bilateral CP (p < 0.001). Further characteristics of the study population are presented in Table 1.

**Table 1:** Characteristics of the study population at the last visit by unilateral and bilateral cerebral palsy subgroup.

Characteristic	Total 389 (100.0)	Unilateral 105 (27.0%)	Bilateral 284 (73.0%)	p
Age in months Mean (SD)	102.1 (53.9)	111.3 (54.6)	98.8 (53.4)	0.100*
Height (cm) Mean (SD)	129.8 (24.8)	135.1 (26.7)	121.1 (23.3)	< 0.001*
Weight (kg) Median (IQR)	23.0(16.0; 36.0)	31.5 (20.6; 50.4)	21.5 (15.5; 30.0)	< 0.001**
BMI (kg/m^2^) Median (IQR)	15.7 (14.2; 18.1)	17.3 (15.4; 20.4)	15.3 (14.0; 17.2)	< 0.001**
Male sex n (%)	226 (58.1)	59 (56.2)	167 (58.8)	0.360***
Prematurity n (%)	151 (38.8)	12 (11.4)	139 (48.8)	< 0.001***
**Gross motor impairment (GMFCS Level)**				
GMFCS* I n (%)	173 (44.5)	94 (89.5)	79 (27.7)	< 0.001****
GMFCS* II n (%)	53 (13.6)	10 (10.5)	43 (15.1)	
GMFCS* III n (%)	58 (14.9)	0 (0.0)	58 (20.4)	
GMFCS* IV n (%)	74 (19.0)	0 (0.0)	74 (26.0)	
GMFCS* IV n (%)	31 (8.0)	0 (0.0)	31 (10.9)	
Dysphagia n (%)	54 (13.9)	1 (0.9)	53 (18.7)	

*GMFCS* Gross Motor Function Classification System.

*t-test, **Mann–Whitney-*U* test.

***Fisher’s exact test.

****Kruskal–Wallis test.

### Height

**Figure 1:**
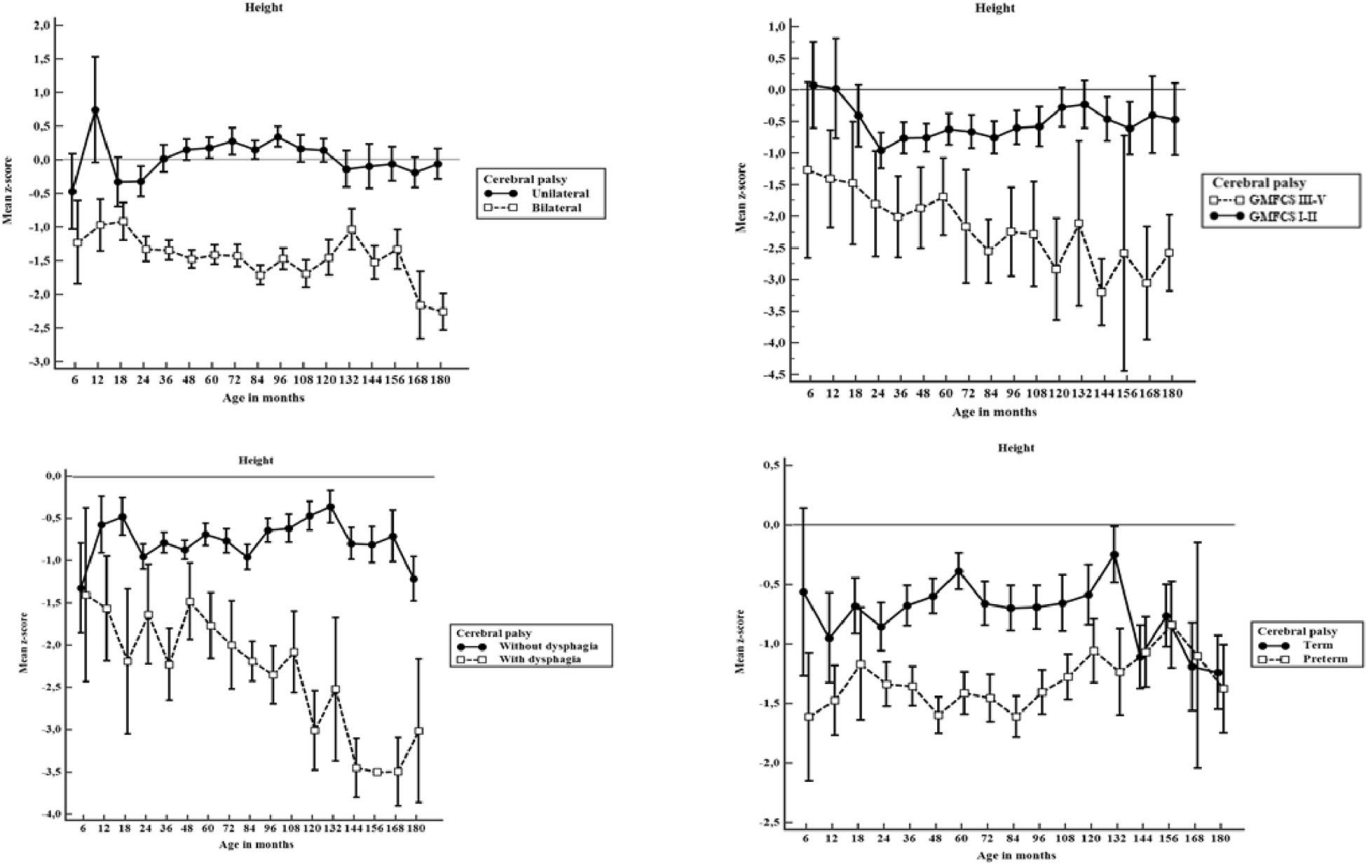
Height-for-age mean z-scores for with 95% CI for height by age in months among children unilateral vs. bilateral cerebral palsy, Gross Motor Function Classification System level I–II vs. III–V, with and without dysphagia and with and without prematurity (n = 1536). If the 95% CI bars overlap, the difference between the two z-score means is not statistically significant (p > 0.05).

Figure 1 compares the mean z-scores for height with 95% CI among children with and without unilateral cerebral palsy, GMFCS level, dysphagia and prematurity.

Children with unilateral CP had near to normal height gain from 6 to 180 months of life with z-scores of height-for-age close to 0. Children with bilateral CP were significantly shorter than children with unilateral CP (p < 0.001).

Children with GMFCS level III–V were significantly shorter than children with bilateral CP GMFCS level I–II (p < 0.001).

Premature children with CP were not significantly different in height from term children with CP between 6 and 18 months (p = 0.117). From 24 to 108 months of life premature children had significantly less growth than term children with CP. After this age, the height growth is similar in both groups. Particularly interesting is the fact that term children with CP had near to normal height gain from 6 to 132 months. But at this age height z-scores dropped when compared to children with CP born premature.

Children without dysphagia had close to normal height gain from 6 to 180 months of life, whereas children with dysphagia were significantly shorter (p < 0.001).

### Weight

**Figure 2:**
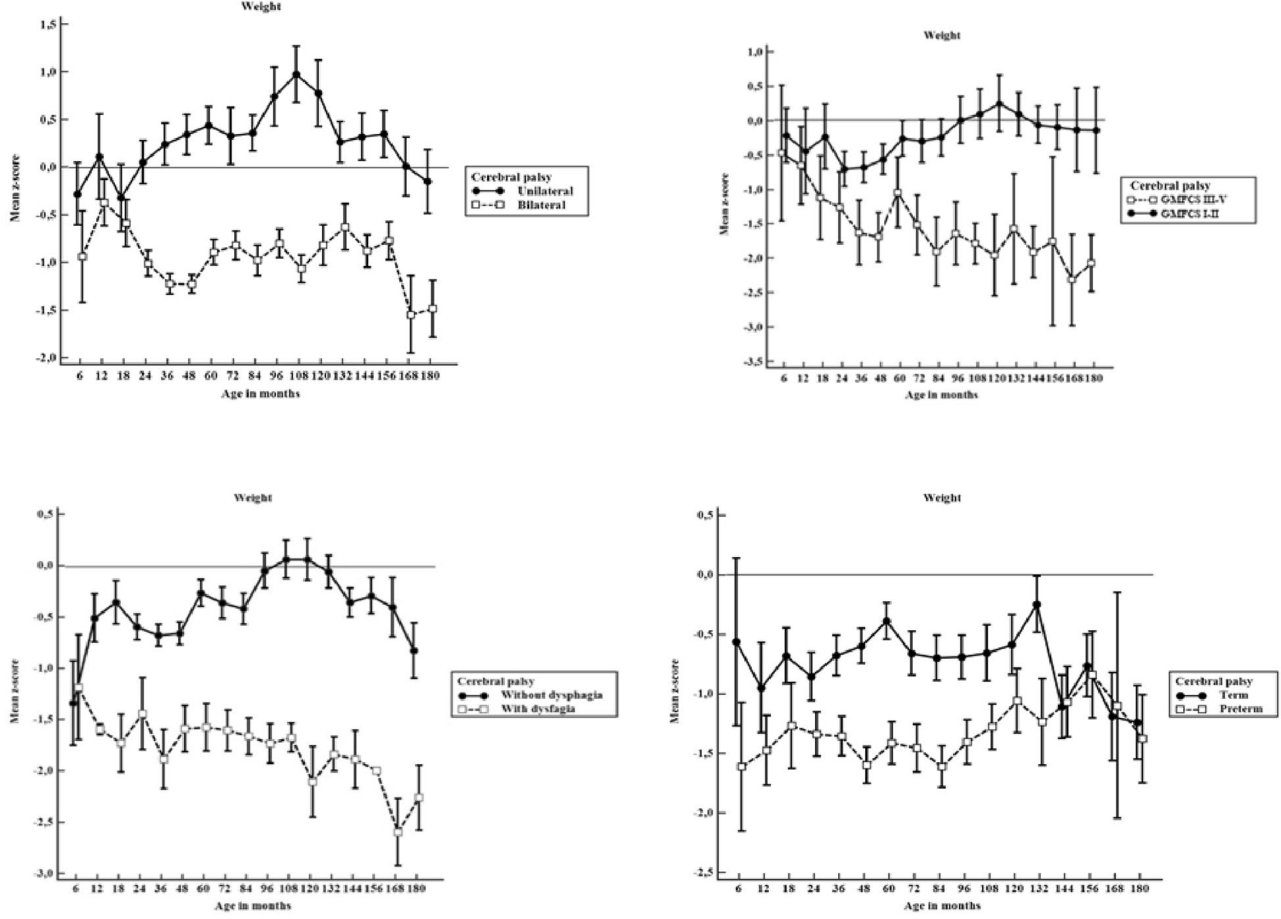
Weight-for-age mean z-scores with 95% CI for weight by age in months among children unilateral vs. bilateral cerebral palsy, Gross Motor Function Classification System level I–II vs. level III–V, with and without dysphagia, and with and without prematurity (n = 1536). If the 95% CI bars overlap, the difference between the two z-score means is not statistically significant (p > 0.05).

Figure 2 displays estimated mean z-scores for weight with 95% CI among children with and without unilateral cerebral palsy, GMFCS level, dysphagia and prematurity.

Children with unilateral CP had near to normal weight gain from 6 to 180 months of life with means of z-scores of weight-for-age of between − 0.5 to + 1. Children with bilateral CP were significantly lighter (p < 0.001).

Considering GMFCS, children with levels I–II had near to normal weight gain with means of z-scores of weight-for-age of between − 0.5 to 0.5, whereas children with levels III–V were significantly lighter (p < 0.001).

Premature children with CP were not significantly different in weight from term children with CP from 6 to 18 months (p > 0.05). From 24 to 108 months of life premature children had significantly less weight than term children with CP. After this age, weight gain is similar in both groups. Parallel to linear growth, term children with CP had near to normal weight gain from 6 to 132 months. But at this age weight z-scores dropped when compared to children with CP born preterm.

Children with dysphagia were significantly lighter than children without this disorder, showing negative z-scores of weight-for-age (p < 0.001).

### Body Mass Index

**Figure 3:**
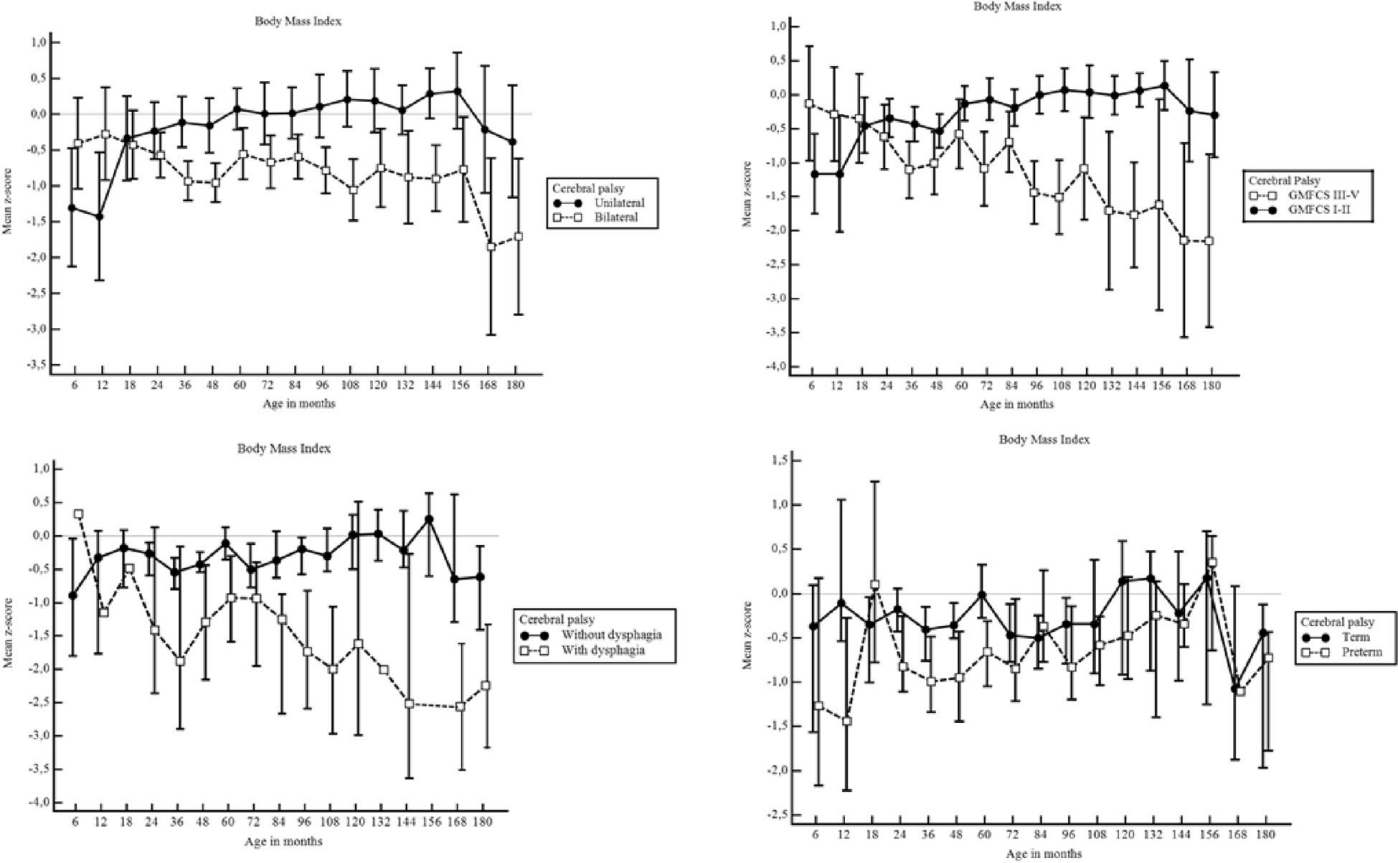
Body Mass Index for age mean z-scores with 95% CI for weight by age in months among children unilateral vs. bilateral cerebral palsy, Gross Motor Function Classification System level I–II vs. level III–V, with and without dysphagia, and with and without prematurity (n = 1536). If the 95% CI bars overlap, the difference between the two z-score means is not statistically significant (p > 0.05).

Figure 3 displays estimated mean z-scores for BMI with 95% CI among children with and without unilateral cerebral palsy, GMFCS level, dysphagia and prematurity.

Children with unilateral CP had near to normal BMI gain from 6 to 180 months of life, with means of z-scores of BMI-for-age of between − 0.5 and 0.5. Children with bilateral CP were significantly lower (p < 0.001). Considering GMFCS, children with levels I–II had near to normal BMI gain with means of z-scores of BMI-for-age of between − 0.5 to 0.5, whereas children with levels III–V had a significantly lower BMI (p < 0.001).

Premature children with CP were not significantly different in BMI from term children with CP from 6 to 180 months (p > 0.05). Children with dysphagia had significantly lower mean BMI z-scores than children without this disorder, (p < 0.001).

### Multivariate analysis

#### Height

In the main effect model, children with bilateral CP, GMFCS level III–V and dysphagia were significantly shorter than children with unilateral CP. Prematurity was not significantly associated with poor height growth in children with bilateral CP compared with children with unilateral CP.

In the interaction effect model, children with bilateral CP and GMFCS level III–V were − 0.943 [− 1.129; − 0.756] z-score units shorter than children with unilateral CP (p < 0.001), plus − 1.360 [− 2.183 to − 0.537] of the bilateral condition alone, resulting in an overall average decrease of − 2.303 in the height z-score. Children with bilateral CP and dysphagia had − 0.545 [− 0.785; − 0.305] z-score units lower z-score of height-for-age than children with unilateral CP (p < 0.001), and remain significantly shorter compared to their peers (Table 2a).

**Table 2:** Mean z-score change for height and weight in children with unilateral and bilateral cerebral palsy (linear mixed models) (n = 1536).

Model	Variables	β	95% CI	p
**(a) Z-score height-for-age from 6 months to 15 years of age**				
Main effect model	Unilateral CP	Ref.		
	Bilateral CP	− 0.953	− 1.145 to − 0.761	**< 0.001**
	GMFCS I–II	− 0.923	− 1.105 to − 0.741	**< 0.001**
	GMFCS III–V	− 1.083	− 1.349 to − 0.905	**< 0.001**
	Dysphagia	− 0.547	− 0.786 to − 0.310	**< 0.001**
	Prematurity	− 0.033	− 0.200 to 0.134	0.70
Interaction effect model	Unilateral CP	Ref.		
	Bilateral CP	− 1.360	− 2.183 to − 0.537	**0.001**
	Bilateral CP + GMFCS III–V	− 0.943	− 1.129 to − 0.756	**< 0.001**
	Bilateral CP + dysphagia	− 0.545	− 0.785 to − 0.305	**< 0.001**
**(b) Z-score weight-for-age from 6 months to 15 years of age**				
Main effect model	Unilateral CP	Ref.		
	Bilateral CP	− 0.999	-− .176 to − 0.807	**< 0.001**
	GMFCS I–II	− 0.571	− 0.745 to − 0.397	**< 0.001**
	GMFCS III–V	− 1.751	− 2.581 to − 1.157	**< 0.001**
	Dysphagia	− 0.635	− 0.864 to − 0.407	**< 0.001**
	Prematurity	− 0.029	− 0.190 to 0.130	0.72
Interaction effect model	Unilateral CP	Ref.		
	Bilateral CP	− 0.904	− 1.697 to − 0.110	**0.026**
	Bilateral CP + GMFCS III–V	− 1.449	− 10.526 to − .677	**0.048**
	Bilateral CP + dysphagia	− 0.629	− 1.189 to − 0.068	**0.028**
**(c) Z-score Body Mass Index-for-age from 6 months to 15 years of age**				
Main effect model	Unilateral CP	Ref.		
	Bilateral CP	− 0.437	− 0.799 to − 0.075	**0.002**
	GMFCS I–II	0.364	0.018 to 0.709	**0.040**
	GMFCS III–V	− 0.364	− 0.709 to − -0.018	**0.040**
	Dysphagia	− 0.902	− 1.340 to − 0.465	**< 0.001**
	Prematurity	− 0.121	− 0.412 to 0.171	0.417
Interaction effect model	Unilateral CP	Ref.		
	Bilateral CP	− 0.482	− 0.835 to − 0.130	**0.007**
	Bilateral CP + GMFCS III–V	− 0.375	− 0.724 to − 0.026	**0.036**
	Bilateral CP + dysphagia	− 0.854	− 1.295 to − 0.414	**< 0.001**

Significant differences are marked in bold letters.

*GMFCS* Gross Motor Function Classification System.

#### Weight

In the main effect model, children with bilateral CP, GMFCS level III–V and dysphagia were significantly lighter than children with unilateral CP. Prematurity was not significantly associated with poor weight gain in children with bilateral CP compared with children with unilateral CP.

In the interaction effect model, children with bilateral CP and GMFCS level III–V remain significantly lighter than children with unilateral CP, decreasing their weight-for-age z-score − 1.449 [− 10.526; − 0.677] units compared to children with Unilateral CP (p < 0.001). Children with bilateral CP and with dysphagia were lighter, growing − 0.629 [− 1.189; − 0.068] z-score units below their weight-for-age compared to children with unilateral CP (p < 0.001) (Table 2b).

#### Body Mass Index

In the main effect model, children with bilateral CP, GMFCS level III–V and dysphagia had a significantly lower BMI than children with unilateral CP. Prematurity was not significantly associated with poor BMI gain in children with bilateral CP compared to children with unilateral CP.

In the interaction effect model, children with bilateral CP, GMFCS level III–V and dysphagia remained with a significantly lower BMI than children with unilateral CP (Table 2c). Children with bilateral CP and GMFCS level III–V decreased their BMI-for-age z-score − 0.375 [− 0.724; − 0.026] units compared to children with Unilateral CP (p = 0.036). Children with bilateral CP and with dysphagia were lighter, growing − 0.854 [− 1.295; − 0.414] z-score units below their BMI-for-age compared to children with unilateral CP (p < 0.001).

## Discussion

In this study we found that poor height and weight growth were associated with bilateral CP phenotype, severity of gross motor impairment levels III to V and dysphagia. In line with a previous report, we found that children with unilateral CP were close to their typically developed peers in normal growth whereas children with bilateral CP already had significantly lower mean height and weight z-scores in infancy, childhood and adolescence^23^.

In previous research, CP phenotypes and GMFCS levels have been independently correlated with more restricted growth^22,24,25^, but it was not yet clear whether there was an association between them that produced more marked stunting and wasting. Other authors reported differences in growth in children with CP associated with feeding ability, motor function, prematurity and born small for gestational age^5,12,23–25^, but they did not analyze them in combination with the CP phenotype. These studies established that growth in children with CP is negatively influenced by feeding, size at birth and motor severity independently. We found that the association between dysphagia and bilateral CP phenotype was strongly predictive for poor growth for height, weight and BMI, and gradually decreased with age, becoming noticeable in adolescence, when it can reach z-scores lower than -2. These findings are in line with other authors who reported that feeding difficulties were predictive of poor growth in children with CP^5,26^.

Preterm birth associated with bilateral CP phenotype in our population study was not a significant predictor of poor growth in height and weight, particularly during infancy and adolescence. During childhood, from 24 to 120 months of age, a difference is observed in height and weight for age z-scores, showing that preterm birth children have significantly lower z-scores. In accordance with this, a previous study follow up until 5 years of life shows that there is a growth difference between children born term and those born preterm, showing restrictive growth in prematurity^12^.

Previous studies have shown that children with CP with growth failure had low basal growth hormone (GH) and Insulin Growth Factor-1 (IGF-1) and responded inadequately to the insulin stimulation test. On the other hand, children with CP with normal growth behaved as typically developed peer controls regarding their basal GH and IGF-1 levels, but failed to respond adequately to the insulin stimulation test. These findings suggest that non nutritional factors contribute to growth retardation in children with CP^27^. For this reason, we hypothesize that during infancy from birth to 24 months, term and preterm PC infants present a similar growth pattern because they have similar IGF-1 levels. While during childhood from 24 to 132 months, preterm children with CP show a significantly lower growth pattern than term children because they have lower GH basal levels. During adolescence, after 132 months of age, children with CP born at term significantly reduce their growth and fall to the level corresponding to those born preterm, possibly because they cannot perform the pubertal growth spurt related to the peak of maximum growth dependent on sex steroids^28^. Future studies are needed to clarify the potential association between growth hormones, sex steroids and growth patterns in children with unilateral and bilateral CP.

The limitations of our study require consideration. A most important limitation is the retrospective study design. Future prospective cohort studies are needed to confirm our findings. Although routinely measured growth data provide an accurate method for data collection in research^29^, in the special case of children with CP, while weight measurements have high reliability, the reliability of height measurements particularly children with severe motor impairments may be less accurate due to contractures or inability to stand upright^4^. To account for this problem, all measures for each child were controlled one by one and we did not include outliers > 5 SD in our data (n = 6).

The strengths of this study include the relatively large number of anthropometric measurements and the representativeness of the children included in this study. Even though this study was limited to a single center, serial growth measurements obtained from a single center are likely to be more reliable than comparisons of growth outcomes across multiple centers.

Growth differences in children with bilateral CP compared with unilateral CP, and motor severity raise interesting questions about the underlying mechanism that leads to reduced growth in each group.

## Conclusion

In conclusion, our data suggest that poor height, weight and BMI growth were associated with bilateral CP phenotype, severity of gross motor impairment levels III to V and dysphagia.

Our study shows the need for additional research on growth rates of children with CP in order to evaluate growth rate differences.

## Abbreviations

CP: Cerebral palsy
CI: Confidence interval
GMFCS: Gross motor function classification system
TD: Typical development
